# *HSPG2* Mutation Association with Immune Checkpoint Inhibitor Outcome in Melanoma and Non-Small Cell Lung Cancer

**DOI:** 10.3390/cancers14143495

**Published:** 2022-07-19

**Authors:** Wenjing Zhang, Zhijuan Lin, Fuyan Shi, Qiang Wang, Yujia Kong, Yanfeng Ren, Juncheng Lyu, Chao Sheng, Yuting Li, Hao Qin, Suzhen Wang, Qinghua Wang

**Affiliations:** 1 Key Laboratory of Medicine and Health of Shandong Province, Department of Health Statistics, School of Public Health, Weifang Medical University, Weifang 261053, China; 2021729@stu.wfmc.edu.cn (W.Z.); shifuyan@wfmc.edu.cn (F.S.); kongyj@wfmc.edu.cn (Y.K.); renyf@wfmc.edu.cn (Y.R.); lvjch@wfmc.edu.cn (J.L.); wangsz@wfmc.edu.cn (S.W.); 2 Key Laboratory for Immunology in Universities of Shandong Province, School of Basical Medicine, Weifang Medical University, Weifang 261053, China; linzhj@wfmc.edu.cn; 3 Department of Epidemiology, School of Public Health, Weifang Medical University, Weifang 261053, China; wangqiang2017@wfmc.edu.cn; 4 National Clinical Research Center for Cancer, Key Laboratory of Molecular Cancer Epidemiology of Tianjin, Department of Epidemiology and Biostatistics, Tianjin Medical University Cancer Institute and Hospital, Tianjin 300060, China; shengchao9505@tmu.edu.cn (C.S.); 13402263808@163.com (Y.L.); 5 Weifang Key Laboratory for Food Nutrition and Safety, School of Public Health, Weifang Medical University, Weifang 261053, China; qinhao981207@wfmc.edu.cn

**Keywords:** *HSPG2* mutations, immunotherapy, melanoma, NSCLC, clinical biomarker, cancer genomics

## Abstract

**Simple Summary:**

Immune checkpoint inhibitors (ICIs) markedly improve the survival benefits of advanced melanoma and non-small cell lung cancer (NSCLC). Nevertheless, only a subset of patients could benefit from such a therapy. Novel and effective clinical biomarkers are needed to assess ICI treatment efficacy. Heparan sulfate proteoglycan 2 (HSPG2) is frequently mutated in melanoma and NSCLC. In this study, we comprehensively integrated the pretreatment somatic mutational profiles and clinical information of both tumors and observed that *HSPG2* mutations were associated with favorable tumor immunogenicity and immunotherapeutic efficacy. Our study provides a potential clinical molecular biomarker for evaluating ICI therapy responses.

**Abstract:**

Immune checkpoint inhibitors (ICIs) markedly promote the survival outcome of advanced melanoma and non-small cell lung cancer (NSCLC). Clinically, favorable ICI treatment efficacy is noticed only in a smaller proportion of patients. Heparan sulfate proteoglycan 2 (HSPG2) frequently mutates in both tumors. Herein, we aim to investigate the immunotherapeutic and immunological roles of *HSPG2* mutations in melanoma and NSCLC. A total of 631 melanoma samples and 109 NSCLC samples with both somatic mutational profiles and clinical immunotherapy data were curated. In addition, by using The Cancer Genome Atlas data, genomic and immunological traits behind *HSPG2* mutations were elucidated. Melanoma patients with *HSPG2* mutations had a markedly extended ICI outcome than other patients. An association between *HSPG2* mutations and the improved outcome was further confirmed in NSCLC. In addition, an elevated ICI response rate was presented in *HSPG2*-mutated NSCLC patients (81.8% vs. 29.7%, *p* = 0.002). Subsequent analyses revealed that *HSPG2*-mutated patients had a favorable abundance of response immunocytes, an inferior abundance of suppression immunocytes, enhanced mutational burden, and interferon response-relevant signaling pathways. We uncovered that *HSPG2* mutations were predictive of a better ICI response and associated with preferable immunogenicity, which may be considered as a genomic determinant to customize biotherapy strategies.

## 1. Introduction

Surgery, radiotherapy, chemotherapy, and targeted therapy are commonly used clinical treatment methods for cancer patients. However, for patients at advanced or metastatic stages, the above treatment modalities may be unsatisfactory [1]. In recent years, the advent of immune checkpoint inhibitors (ICIs) has greatly prolonged the prognosis of cancer patients [2]. The main theory of ICI treatments is to battle tumor cells by activating the immune system [2]. ICI agents have become the clinical first-line treatment strategy for several cancers; nevertheless, their biggest drawback is that only a small percentage of patients can benefit from them [3]. Therefore, selecting a suitable population to receive such ICI treatments is necessary.

At present, multiple biomarkers are determined to evaluate cancer immunotherapeutic efficacy. Programmed cell death 1 ligand 1 (PD-L1) is the first approved molecular biomarker for predicting anti-PD-1/L1 treatment response [4]. Its elevated expression was demonstrated to connect with favorable ICI efficacy [5]. However, in several clinical trials, PD-L1-negative tumors could also benefit from immunotherapy [6]. Tumor mutation burden (TMB) was recently reported to be involved in a better clinical immune therapy outcome [7]. Inconsistent results derived from several studies [8] showed that tumors with high TMB did not exhibit the treatment benefits. The above evidence demonstrated that PD-L1 expression and TMB sometimes are imperfect in predicting immunotherapeutic effects. Recently, multiple novel ICI biomarkers were reported, including gene mutations (e.g., *POLE* [9], *JAK1/2* [10], *B2M* [10], and *MUC16* mutations [11]), specific mutational signatures (e.g., signatures 1, 4, 7, and 11 [12]), and molecular subtypes [13].

Heparan sulfate proteoglycan 2 (HSPG2) encodes the perlecan protein, which comprises a central protein to which three long chains of glycosaminoglycans (heparan sulfate or chondroitin sulfate) are attached. It has been revealed that the perlecan protein exhibits vital roles in multiple biological behaviors via the interaction with prolargin, laminin, collagen type IV, transthyretin, etc. Several recent studies have demonstrated that HSPG2 overexpression was associated with invasion, metastasis, and an inferior survival outcome in triple-negative breast cancer [14], acute myeloid leukemia [15], glioblastoma [16,17], oligoastrocytoma [18], and oligodendroglioma [18]. HSPG2 was also reported to regulate immune and stromal infiltration in glioma [19] and prostate cancer [20]. Lima et al. performed a proteogenomics analysis and found that HSPG2-specific mutations played a protective role in prostate cancer [21]. So far, no studies have revealed the correlation of *HSPG2* mutations with immunological features and ICI treatment efficacy in cancers.

Taking into account that ICI treatments are most commonly used for melanoma and NSCLC patients, in this work, we comprehensively integrated the pretreatment somatic mutational profiles from melanoma and NSCLC; furthermore, clinical information after immunotherapy of both tumors was also obtained. Finally, based on 631 melanoma and 109 NSCLC samples, we investigated the immunological and clinical immunotherapeutic implications of *HSPG2* alterations. This immunogenomic research might provide useful clues for customizing cancer immunotherapy strategies.

## 2. Materials and Methods

### 2.1. Samples Used in This Study

From previous publications, we integrated a total of 631 melanoma [22,23,24,25,26,27,28,29] and 109 NSCLC samples [30,31] with both somatic mutational profiles and ICI treatment information. All included samples in this study were treated with blockade treatment of immune checkpoints (i.e., PD-1/L1, CTLA-4, or combination). Since genomic mutation data were acquired from distinct sequencing and annotating platforms, we re-annotated them with the Oncotator software (developed by Ramos et al., Boston, MA, USA) against the h19 reference genome [32]. In this research, nonsynonymous mutations (i.e., missense mutations, nonsense mutations, frameshift del/ins, in frame del/ins, and splice site mutations) were employed for subsequent analyses. The detailed clinical baseline characteristics and ICI therapy information for melanoma and NSCLC samples are shown in Table S1 and Table S2, respectively.

**Figure 1:**
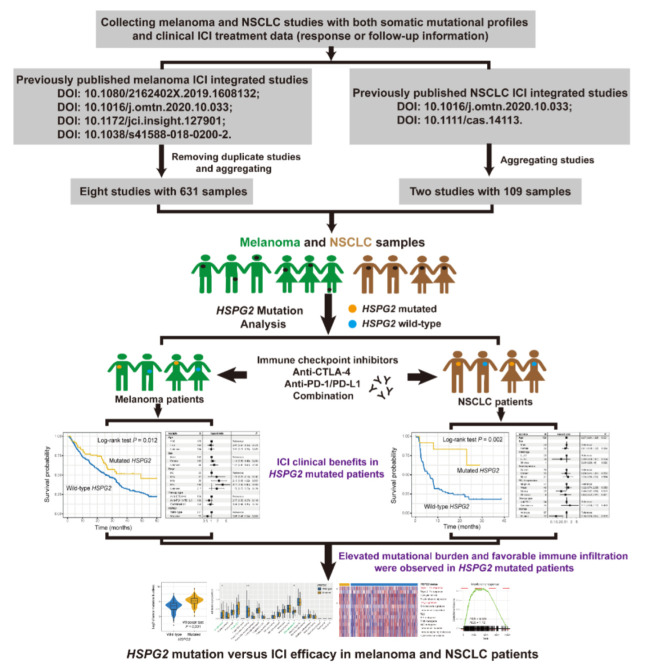
Workflow of this study. Investigation of the roles of *HSPG2* mutations in evaluating ICI treatment efficacy in melanoma and NSCLC.

From The Cancer Genome Atlas (TCGA) project (http://xena.ucsc.edu/ accessed on 1 May 2022), we obtained a total of 457 melanoma and 995 NSCLC samples with genomic mutation data, transcriptomic expression profiles, and clinical information. Especially, the log2 transformed and normalized gene expression profiles of both tumors were applied to explore the potential immunological mechanisms behind *HSPG2* mutations. The detailed flowchart of this research is shown in Figure 1.

### 2.2. Mutational Signatures in the Genome

Mutational signatures were extracted using mutational profiles of melanoma and NSCLC samples based on a method proposed by a recent study [33]. In this method, Bayesian nonnegative matrix factorization (NMF) was used to disassemble mutation feature matrix *A* with 96 base substitution types into 2 nonnegative matrices *W* and *H* (i.e., *A* ≈ *W* × *H*), with *W* indicating the detected specific mutational signatures and *H* representing the mutational activities for each signature. The number of columns of matrix *W* is the number of mutational signatures. The rows of matrix *A* are the 96 mutational contexts, and its columns are the integrated samples of both cohorts. The 96 mutational contexts are derived from combinations of 6 mutational types (i.e., C > A, C > G, C > T, T > A, T > C, and T > G) and their 5′ and 3′ adjacent bases. The rows and columns of matrix *H* indicate the individual signatures and their corresponding mutational activities, respectively. The pruning process is performed by introducing the weight parameter λ_k_, which is associated with the *k*th column of *W* and the *k*th row of *H*. All extracted mutational signatures were then compared with well-annotated signatures stored in the COSMIC database (version 2, Cambridge, UK) using cosine similarity.

### 2.3. Tumor-Infiltrating Immune Cells

To elucidate the different immune cell infiltrating abundances between *HSPG2* mutant and wild-type groups, we employed CIBERSORT and the Angelova et al. algorithm to evaluate infiltrating levels of distinct immunocytes. CIBERSORT uses the LM22 signature, which includes 547 representative genes to assess tumor-infiltrating levels of 22 immunocytes [34]. The Angelova et al. algorithm applies a feature signature with 812 genes to evaluate the infiltration abundance of 31 immunocytes [35]. The detailed characteristic genes for each immune cell subtype are collected in Table S3.

### 2.4. Immune Infiltration and Immunogenicity-Related Signatures

Recent research presented multiple molecular signatures associated with immune infiltration and tumor immunogenicity. We thus curated the relevant signatures as follows: (1) immune/stromal cell signatures [36]; (2) an immune cell subset of T cells, B cells, and natural killer (NK) cells [37]; (3) T/NK cells, B/plasma cells, and monocyte/dendritic cell enrichment signature [38]; (4) Type 1/2 interferon (IFN) signature [39]; (5) IFNγ signature [40]; (6) T cell-inflamed gene expression profile (GEP) [41]; (7) immune cytolytic activity [39]; (8) immune signaling molecules [37]; (9) cytokines and chemokines [37]; and (10) tertiary lymphoid structures [42]. Detailed characteristic genes for distinct immunogenicity signatures are illustrated in Table S4.

### 2.5. GSVA and GSEA

Single sample gene set enrichment analysis (ssGSEA) was utilized to evaluate enriched levels of collected immunocyte- and immunogenicity-relevant signatures under the GSVA R package [43]. R DESeq2 package [44] was used to perform whole-genome differential analysis between *HSPG2*-mutant and wild-type groups. All *t* values extracted from the differential result were then considered as the input variable to conduct GSEA and acquire specific biological circuits of *HSPG2* mutations. Hallmark pathways were applied as the background comparison circuits.

### 2.6. TMB and NB

Tumor mutation burden (TMB) was defined as the log2 transition of total non-synonymous mutations per megabase in both integrated and TCGA datasets. Neoantigen burden (NB) was calculated according to a recent method [45] for 224 melanoma and 109 NSCLC-integrated samples. From the Cancer Immunome Atlas (TCIA), we acquired the neoantigen data of 340 melanoma and 656 NSCLC samples in the TCGA cohort.

### 2.7. Statistical Analysis

R software was utilized to conduct relevant analyses. Mutational features of specified genes were exhibited with a waterfall plot, which is embedded in the maftools package [46]. The pheatmap package was employed to achieve heatmap exhibition of different immunogenicity signatures in two *HSPG2* subgroups. Survival plots were obtained using the Kaplan–Meier method, and the Logrank test was applied to evaluate significant differences. Multivariate logistic and Cox regression models with multiple confounders taken into consideration were performed using the forest model package. The relationships of continuous and categorical factors with *HSPG2* mutations were estimated with the Wilcoxon rank sum test and Fisher’s exact test, respectively. The detailed sample size and cohorts used for specific *HSPG2*-related analyses are shown in Table S5.

## 3. Results

### 3.1. HSPG2 Mutations of Melanoma

Among the 631 pooled melanoma samples, 193 (30.6%) exhibited the ICI status of complete response (CR) or partial response (PR); 430 (68.1%) had the response of stable disease (SD) or progressive disease (PD), and immunotherapy response data of the remanent samples (1.3%) were not available. The mutational landscape of these melanoma samples was characterized by C > T substitutions (Figure S1). Mutational features of significantly mutated melanoma genes concerning *HSPG2* mutations are illustrated in Figure S1. *HSPG2* is frequently mutated, accounting for 80 of 631 patients (12.7%). Amino acid changes produced by *HSPG2* alterations are illustrated with a lollipop plot (Figure S2).

### 3.2. HSPG2 Mutations Associated with Melanoma ICI Outcome

**Figure 2:**
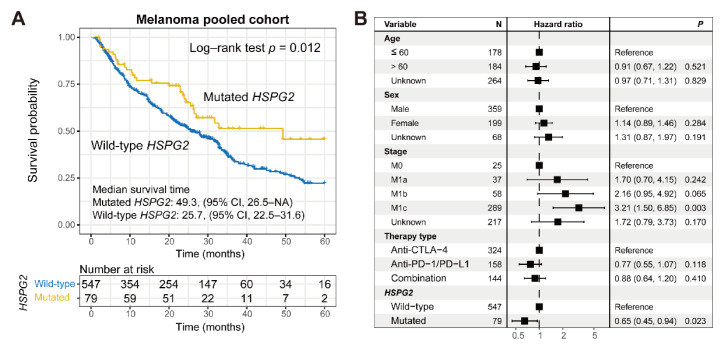
Association of *HSPG2* mutations with ICI treatment outcome in melanoma. (**A**) Kaplan-Meier survival curves of melanoma patients with and without *HSPG2* mutations. (**B**) Representation of multivariate Cox regression model of *HSPG2* mutations with multiple confounding factors adjusted.

A melanoma univariate prognosis analysis revealed that patients with *HSPG2* mutations presented a significantly more prolonged outcome than *HSPG2* wild-type patients (median survival time: 49.3 vs. 25.7 months, Log–rank test *p* = 0.012; Figure 2A). Multivariate Cox regression analysis adjusted for age, sex, stage, and treatment type further corroborated this connection (HR: 0.65, 95% CI: 0.45–0.94, *p* = 0.023; Figure 2B). ICI predictive implications of *HSPG2* mutations in the included individual cohorts and distinct therapy types are exhibited in Figure S3 and Figure S4, respectively.

### 3.3. Connection of HSPG2 Mutations with Mutational Burden in Melanoma

**Figure 3:**
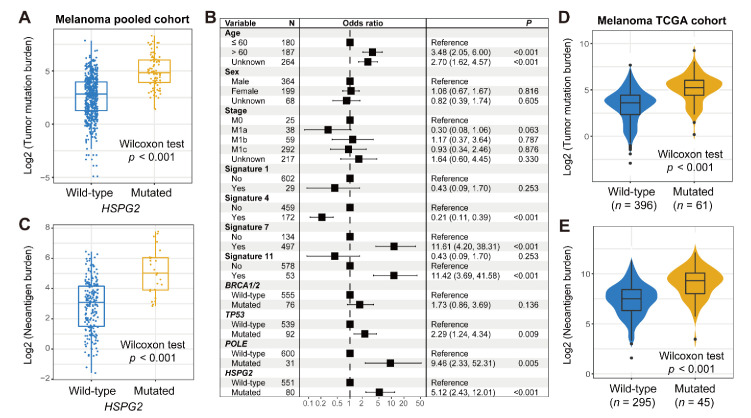
Association of *HSPG2* mutations with mutational burden in melanoma. (**A**) Distinct TMB distribution in *HSPG2*-mutated versus wild-type subgroups. (**B**) Multivariate logistic regression model of *HSPG2* mutations was performed with clinical and genomic confounders taken into consideration. (**C**) Distinct NB distribution in *HSPG2*-mutated versus wild-type subgroups. Connection of *HSPG2* mutations with (**D**) TMB and (**E**) NB based on the data from the TCGA melanoma cohort.

TMB was recently identified as a promising marker to evaluate immune treatment efficacy in advanced cancers. We thus investigated the correlation between *HSPG2* mutations and melanoma TMB. The results show that *HSPG2*-mutated patients harbored a markedly more elevated TMB than wild-type patients (Wilcoxon rank sum test *p* < 0.001; Figure 3A). Genomic mutational signatures play vital roles in regulating genome stability. Based on melanoma mutational profiles, we determined a total of four mutational signatures by employing the NMF method; they are signatures 1, 4, 7, and 11. Specific mutational activities of the above four signatures for each patient are curated in Table S6. We subsequently performed a multivariate logistic model by incorporating clinical features and determined mutational signatures and DNA repair gene mutations to acquire an accurate connection between *HSPG2* mutations and TMB. The results demonstrate that the connection of *HSPG2* mutations with TMB was still existent in the multivariate analysis (OR: 5.12, 95% CI: 2.43–12.01, *p* < 0.001; Figure 3B). In addition, a higher NB was also enriched in *HSPG2*-mutated patients (Wilcoxon rank sum test *p* < 0.001; Figure 3C). We also used the mutational profiles of melanoma samples from the TCGA cohort and observed a significantly elevated TMB and NB in *HSPG2*-mutated patients (Wilcoxon rank sum test both *p* < 0.001; Figure 3D,E).

### 3.4. Validation in NSCLC

**Figure 4:**
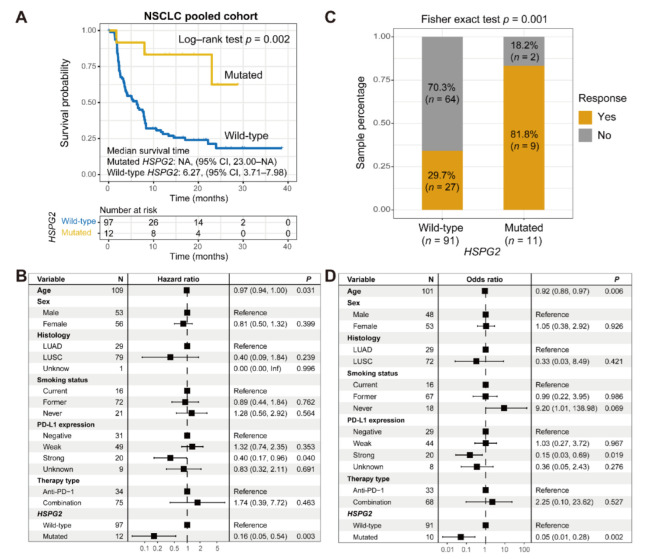
Association of *HSPG2* mutations with ICI treatment efficacy in NSCLC. (**A**) Kaplan–Meier survival curves of NSCLC patients with and without *HSPG2* mutations. (**B**) Representation of multivariate Cox regression model of *HSPG2* mutations with multiple confounding factors adjusted. (**C**) ICI response rate exhibition of *HSPG2*-mutated and wild-type groups. (**D**) Multivariate logistic regression model of *HSPG2* mutations was performed with clinical and genomic variables taken into consideration.

Among the 109 collected NSCLC patients, 36 (33.0%) harbored the CR/PR immunotherapeutic responses. *HSPG2* was also frequently mutant in NSCLC, accounting for 12 of 109 (11.0%) patients. A prognosis analysis showed that *HSPG2* mutations predicted a significantly improved ICI outcome in NSCLC (Log–rank test *p* = 0.002; Figure 4A). We further conducted a multivariate-adjusted Cox regression analysis with multiple confounding factors (e.g., age, sex, smoking status, histology, and PD-L1 expression) taken into consideration, and the consistent association between *HSPG2* mutations and the favorable outcome was also observed (HR: 0.16, 95% CI: 0.05–0.54, *p* = 0.003; Figure 4B). ICI prognostic implications of *HSPG2* mutations in distinct treatment types are illustrated in Figure S5. An immunotherapeutic response analysis revealed that an enhanced ICI response rate was enriched in patients with *HSPG2* mutations (81.8 vs. 29.7%, Fisher’s exact test *p* = 0.001; Figure 4C). Consistently, in a multivariate logistic analysis, this link was still significant after adjusting for other variables (OR: 0.05, 95% CI: 0.01–0.28, *p* = 0.002; Figure 4D).

**Figure 5:**
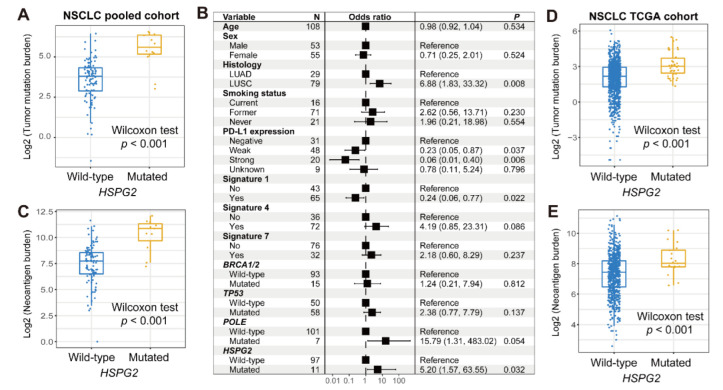
Association of *HSPG2* mutations with mutational burden in NSCLC. (**A**) Distinct TMB distribution in *HSPG2*-mutated versus wild-type subgroups. (**B**) Multivariate logistic regression model of *HSPG2* mutations was performed with clinical and genomic confounders taken into consideration. (**C**) Distinct NB distribution in *HSPG2*-mutated versus wild-type subgroups. Connection of *HSPG2* mutations with (**D**) TMB and (**E**) NB based on the data from the TCGA NSCLC cohort.

We further explored the association of *HSPG2* mutations with the mutational burden in NSCLC. The results show that a markedly higher TMB was observed in patients with *HSPG2* mutations (Wilcoxon rank sum test *p* < 0.001; Figure 5A). Mutational signatures 1, 4, and 7 in NSCLC mutational profiles were extracted, and their mutational activities are exhibited in Table S7. A multivariate-adjusted logistic model with multiple confounding factors taken into account was conducted, and the results indicate that the higher TMB was still enriched in *HSPG2*-mutated patients (OR: 5.20, 95% CI: 1.57–63.55, *p* = 0.032; Figure 5B). In addition, *HSPG2* mutations were also identified to be linked with an elevated NB (Wilcoxon rank sum test *p* < 0.001; Figure 5C). In the TCGA NSCLC cohort, consistently, the associations of *HSPG2* mutations with higher TMB and NB were also noticed (Wilcoxon rank sum test both *p* < 0.001; Figure 5D,E).

### 3.5. Immunologic Features behind HSPG2 Mutations

**Figure 6:**
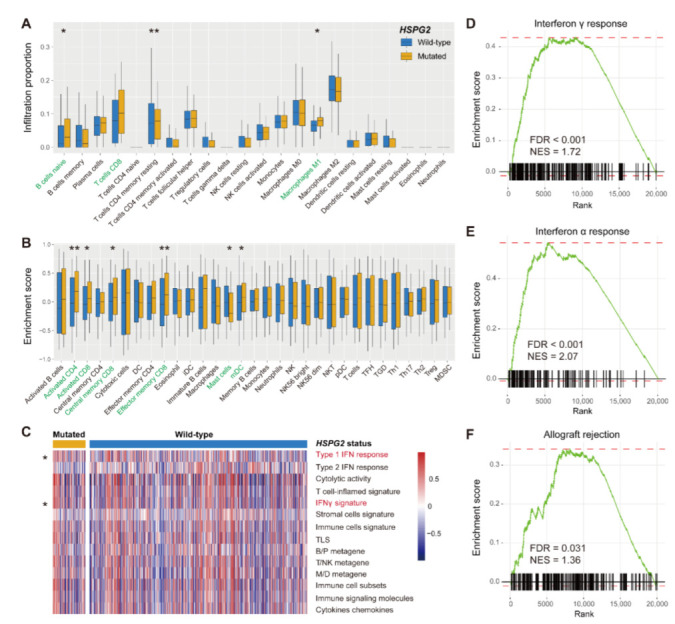
Immunocyte infiltration and signaling circuits behind *HSPG2* mutations in melanoma. (**A**) CIBERSORT method inferred the distinct infiltrating levels of 22 immune cells based on *HSPG2* mutational status. Immune cells highlighted with green are significantly differentially infiltrated. (**B**) Angelova et al. algorithm inferred the distinct infiltrating levels of 31 immune cells based on *HSPG2* mutational status. (**C**) Distinct enrichment distribution of 14 curated immune signatures in *HSPG2*-mutated versus wild-type subgroups. Signatures highlighted with red are significantly differentially enriched. (**D**–**F**) Significantly enriched signaling pathways connected with *HSPG2* mutations. * *p* < 0.05, ** *p* < 0.01.

In melanoma, we performed analyses of immune infiltration, immunogenicity signatures, and pathway enrichment to elucidate the potential immunological mechanisms of *HSPG2* mutations. The CIBERSORT method revealed that the significantly increased infiltration levels of CD8 T cells, M1 macrophages, and B naive cells were enriched in the *HSPG2-* mutated subgroup (all *p* < 0.05; Figure 6A). According to the results from the Angelova et al. algorithm, activated CD4/8 T cells, central/effector memory CD8 T cells, and dendritic cells exhibited an enhanced infiltration in *HSPG2*-mutated patients (all *p* < 0.05; Figure 6B); however, a decreased infiltration of mast cells, which were recently reported to associate with immune suppression, was observed in these mutated patients (*p* < 0.05; Figure 6B).

We then composed a heatmap with distinct immune signature enrichment in two *HSPG2* groups (Figure 6C). We found that the type I interferon response and interferon gamma signatures were significantly presented in *HSPG2*-mutated patients (both *p* < 0.05). Further GSEA analysis verified the results that interferon gamma and alpha responses were enriched in patients with *HSPG2* mutations (both FDR < 0.001; Figure 6D,E and Figure S6). In addition, the immune response-related circuit of allograft rejection also appeared in the *HSPG2-*mutated subgroup (FDR = 0.031; Figure 6F and Figure S6).

Subsequently, we investigated the immune infiltration status of *HSPG2* mutations in NSCLC. Consistent with the findings for melanoma, the markedly increased infiltration levels of immune response cells (e.g., central memory CD4/8 T cells, effector memory CD8 T cells, and cytotoxic cells) and decreased infiltration levels of immune suppressive cells (e.g., regulatory T cells) were observed in NSCLC patients harboring *HSPG2* mutations (all *p* < 0.05; Figure S7A,B).

## 4. Discussion

Immune checkpoint blockade agents promote survival for cancer patients; however, an obvious disadvantage of such a treatment is the lower response rate. Therefore, identifying patients who are suitable to receive ICI treatments is clinically necessary. Immunotherapies are commonly used for melanoma and NSCLC; in this study, by integrating somatic mutational profiles and clinical therapy information for the above two cancers, we determined that *HSPG2* mutations were predictive of favorable immunogenicity and ICI efficacy. The findings obtained from this work would provide a potential molecular biomarker for evaluating immunotherapeutic efficacy.

Activated, central/effector memory CD4/8 T cells were previously demonstrated to play a positive role in promoting tumor immune responses [47,48]. Two macrophage subtypes (i.e., M1 and M2) exhibit inverse immune regulation functions, with M1 macrophages associating with immune response and M2 associating with immune suppression [49,50]. Regulatory T cells are classical immune suppressive cells and mediate the tumor immune escape [51]. Mast cells, an immunocyte subtype, played distinct roles (i.e., immune promotion and inhibition) under distinct signaling regulation [52]. Recently reported immunocyte infiltration evaluation methods (e.g., CIBERSORT and Angelova et al.) give us a chance to depict the immune infiltration landscape across all included samples and to investigate the association between *HSPG2* mutations and specific immune cell infiltration. In this study, we observed that an elevated infiltration of immune response cells (e.g., CD8 T cells and M1 macrophages) and a decreased infiltration of immune suppressive cells (e.g., regulatory T cells and mast cells) were enriched in *HSPG2-* mutated patients, which suggests that *HSPG2* mutations mediate a favorable immune infiltration and tumor microenvironment.

Multiple studies have revealed the potential implications of TMB for evaluating cancer immunotherapeutic efficacy [8,12,13,53]. However, in clinical practice, the determination of TMB needs to conduct whole-exome mutational detection [54]. Another disadvantage of the TMB application is the uncertain cut-off values in distinct cancer types [54]. Therefore, easier surrogates are necessary for such clinical settings. Recent research has shown that mutations in single genes, such as *POLE* [9], *NLRP3* [55], *COL3A1* [12], and *PTPRT* [54] could predict tumor TMB and immunotherapeutic response. In this study, *HSPG2* mutations were also determined to link with an elevated mutational burden and a preferable ICI efficacy in melanoma and NSCLC, which indicates that *HSPG2* mutations may be a possible indicator for TMB and cancer immune treatment response.

The findings derived from this work show that *HSPG2* mutations were connected with an improved outcome in melanoma and NSCLC patients under an immunotherapy setting. We then investigated whether *HSPG2* mutations play roles in the above two cancers treated with conventional chemotherapies in the TCGA cohort. The results demonstrate that no significant associations between *HSPG2* mutations and patient prognosis were observed in both two tumors (multivariate-adjusted both *p* > 0.05; Figure S8). These findings suggest that *HSPG2* mutations play a predictive role regarding cancer immunotherapeutic efficacy rather than a prognostic role.

In this study, based on the integrated ICI-treated 631 melanoma and 109 NSCLC samples, we observed that *HSPG2* mutations were predictive of a favorable ICI treatment outcome, which provides evidence for customizing immunotherapeutic strategies. By using the same pooled melanoma and NSCLC cohorts [12,54,56], we also discovered other mutations of the genes *FAT1*, *COL3A1*, *NRAS*, *NARS2*, *DCC*, and *PTPRT* were associated with better ICI response and outcome. In addition, the ICI efficacy-related mutational signatures and molecular subtypes were also determined. The above findings emphasize the importance of clinically expanded cohorts to uncover novel molecular determinants of response to immunotherapies.

There are several limitations to this study. First, the integration of melanoma and NSCLC data was based on multiple distinct datasets, which might produce some biases during data analyses. Second, only two cancer types were included in this study, and no more available cancers were used for validation. Three, the connection between *HSPG2* mutations and ICI efficacy lacked functional experiments and in-house verification.

## 5. Conclusions

Collectively, based on the aggregated melanoma and NSCLC immunotherapeutic cohorts, we discovered that *HSPG2* mutations were associated with better tumor immunogenicity and ICI treatment efficacy. The results from this genomic association study suggest that *HSPG2* mutations may be considered as a possible molecular biomarker for assessing immune treatment responses.

## Data Availability

Genomic and clinical data used in this study were obtained from previously published studies and can be obtained by contacting the corresponding author under reasonable requests. The codes used for reproducing the results of this study can be acquired by contacting the first authors.

## Supplementary Materials

The following supporting information can be downloaded at: https://www.mdpi.com/article/10.3390/cancers14143495/s1, Figure S1: Mutational patterns of *HSPG2* and common melanoma driver genes illustrated with waterfall plot; Figure S2: Detailed amino acid changes induced by *HSPG2* mutations in the integrated melanoma cohort; Figure S3: Kaplan-Meier survival analyses of *HSPG2* mutations in individual ICI-treated melanoma cohorts; Figure S4: Kaplan-Meier survival analyses of *HSPG2* mutations in distinct ICI treatment types in melanoma; Figure S5: Kaplan-Meier survival analyses of *HSPG2* mutations in individual ICI-treated NSCLC cohorts; Figure S6: Significantly enriched signaling pathways in *HSPG2-*mutated subgroups in melanoma. Immune response pathways are highlighted with green; Figure S7: Immune infiltration associated with *HSPG2* mutations in NSCLC. (A) Distinct infiltration of 22 immunocytes of *HSPG2-*mutated and wild-type groups evaluated with CIBERSORT algorithm. Immunocytes highlighted with green are significantly differentially infiltrated. (B) Distinct infiltration of 31 immunocytes of two *HSPG2* groups evaluated with Angelova et al. method; Figure S8: Prognostic capacities of *HSPG2* mutations in (A) melanoma and (B) NSCLC patients derived from the TCGA project; Table S1: Clinical characteristics of 631 pooled melanoma patients treated with ICI agents; Table S2: Clinical characteristics of 109 pooled NSCLC patients treated with ICI agents; Table S3: Specific genes used for enrichment analysis in each infiltrating immune cell subtype; Table S4: Specific genes used for enrichment analysis in each curated immune-related signature; Table S5: Sample size and cohorts for specific *HSPG2*-related analyses in this study; Table S6: The extracted 4 mutational signatures and their mutational activities in the pooled melanoma cohort; Table S7: The extracted 3 mutational signatures and their mutational activities in the pooled NSCLC cohort.
