# Supplementary material for: HSPG2 Mutation Association with Immune Checkpoint Inhibitor Outcome in Melanoma and Non-Small Cell Lung Cancer
Source: Cancers (Basel). 2022 Jul 19;14(14):3495. doi: 10.3390/cancers14143495 (PMC9315784; doi:10.3390/cancers14143495)
Supplement: Supplementary file 1 [file cancers-14-03495-s001.zip › Supplementary Figures.pdf]

Supplementary Figures

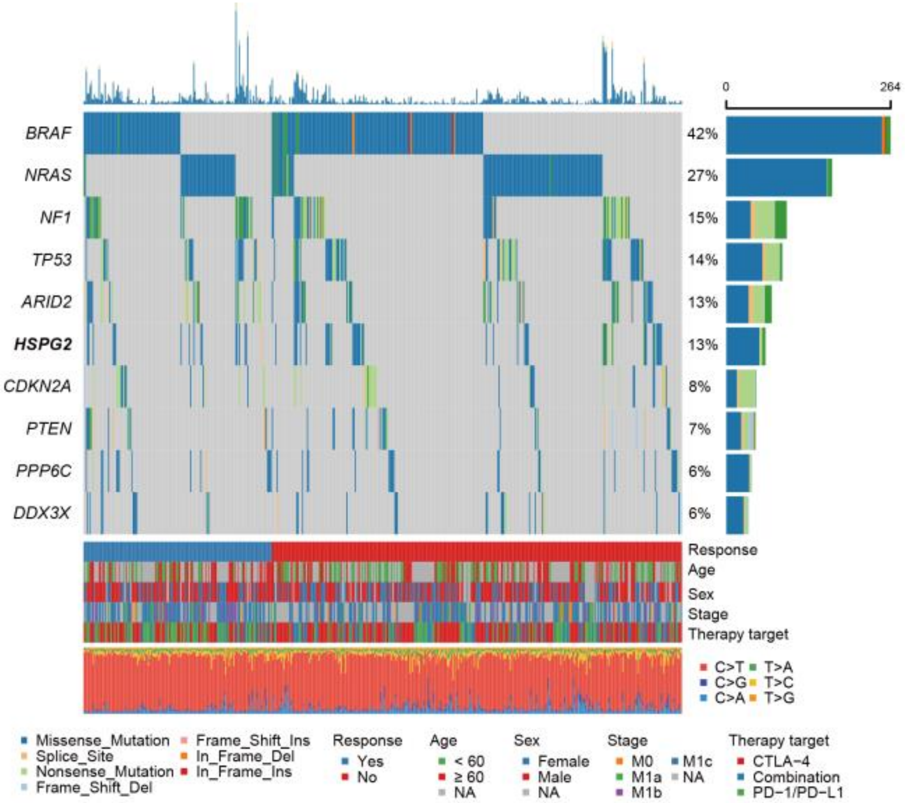

Figure S1. Mutational patterns of *HSPG2* and common melanoma driver genes illustrated with waterfall plot.

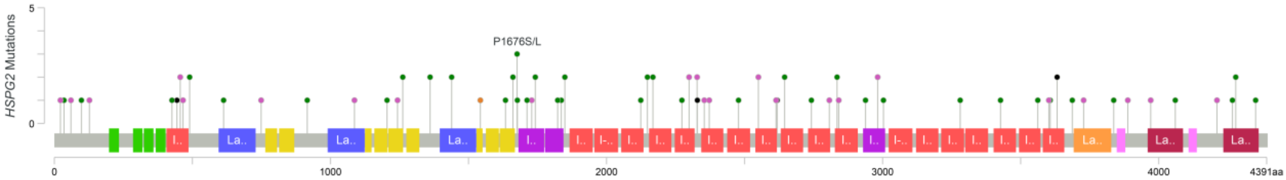

Figure S2. Detailed amino acid changes induced by *HSPG2* mutations in the integrated melanoma cohort.

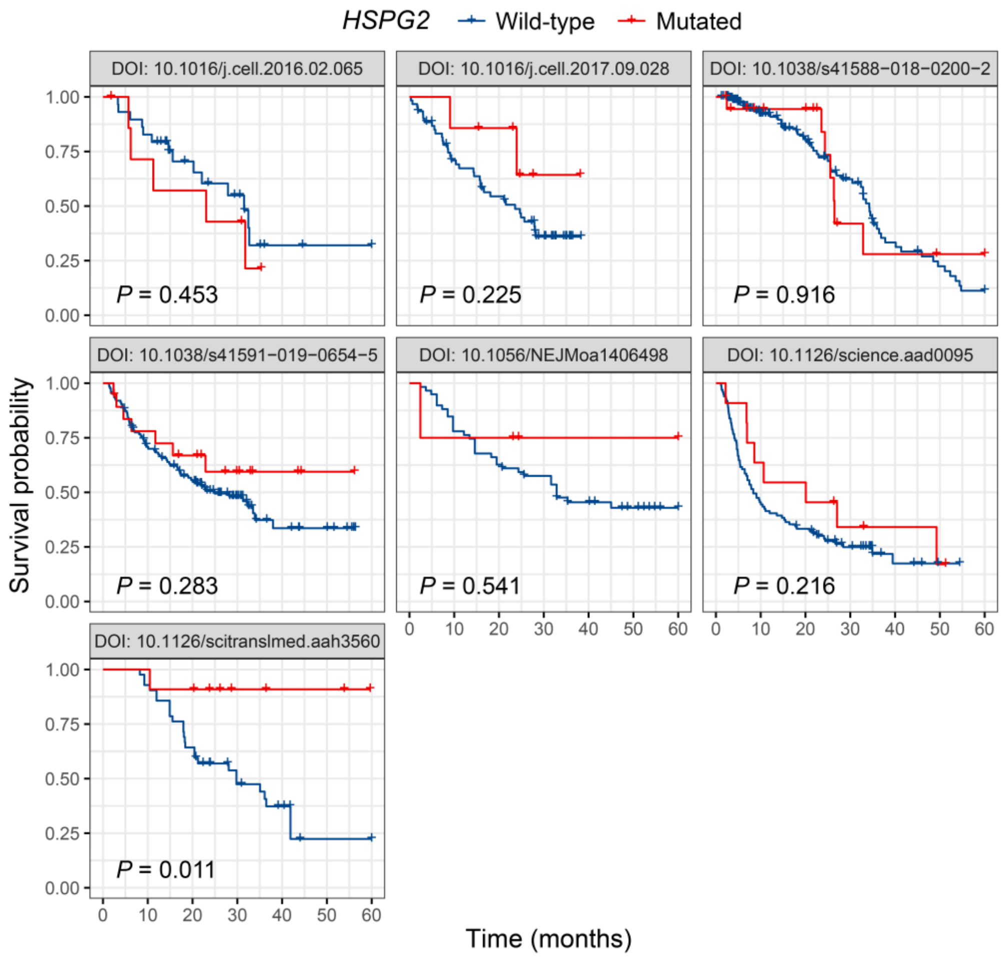

Figure S3. Kaplan-Meier survival analyses of *HSPG2* mutations in individual ICI-treated melanoma cohorts.

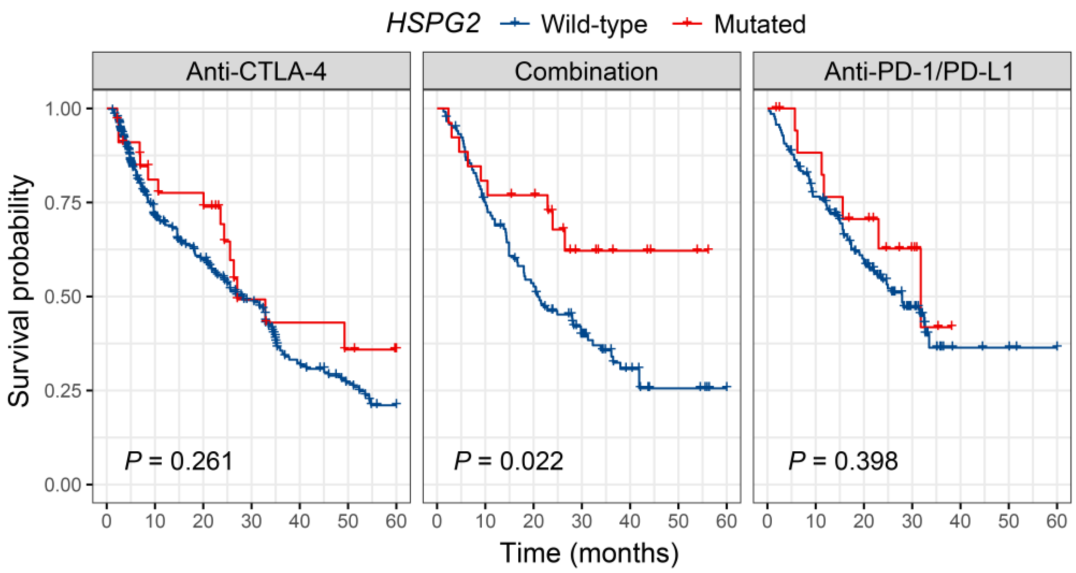

Figure S4. Kaplan-Meier survival analyses of *HSPG2* mutations in distinct ICI treatment types in melanoma.

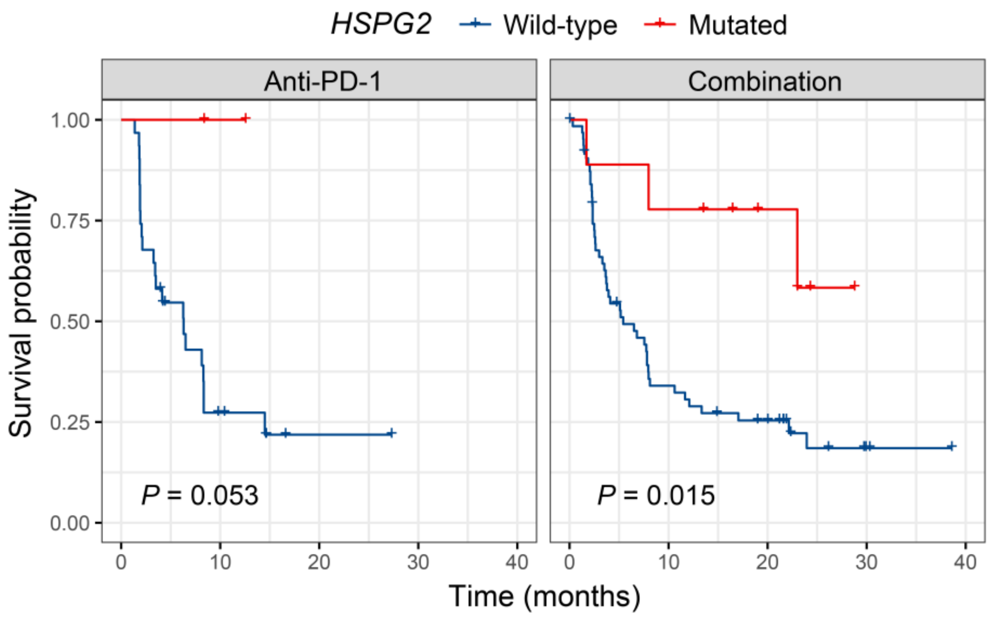

Figure S5. Kaplan-Meier survival analyses of *HSPG2* mutations in individual ICI-treated NSCLC cohorts.

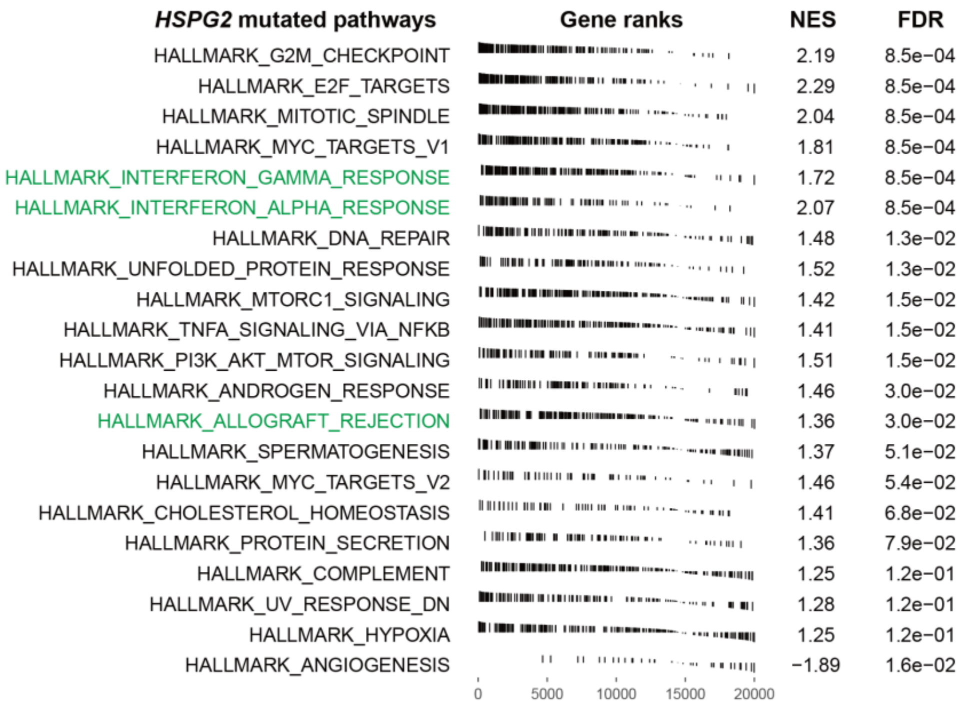

Figure S6. Significantly enriched signaling pathways in *HSPG2* mutated subgroups in melanoma. Immune response pathways were highlighted with green.

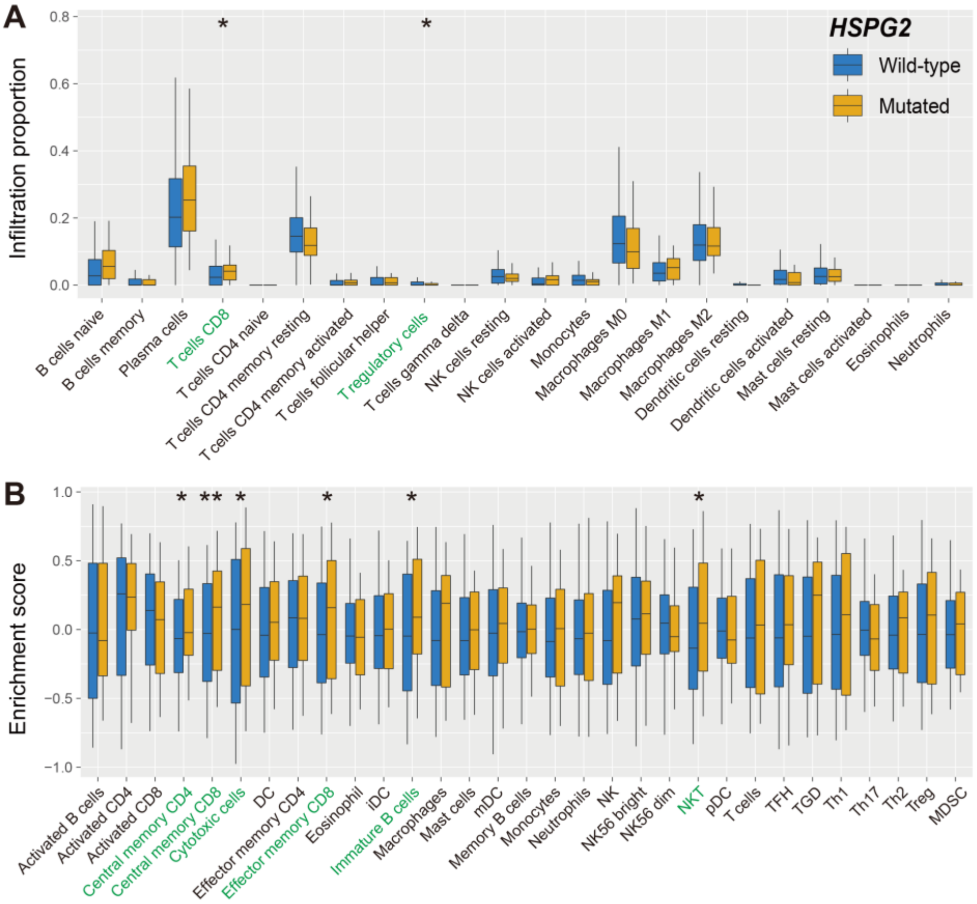

Figure S7. Immune infiltration associated with *HSPG2* mutations in NSCLC. (A) Distinct infiltration of 22 immunocytes of *HSPG2* mutated and wild-type groups evaluated with CIBERSORT algorithm. Immunocytes highlighted with green are significantly differentially infiltrated. (B) Distinct infiltration of 31 immunocytes of *HSPG2* two groups evaluated with Angelova *et al.* method.

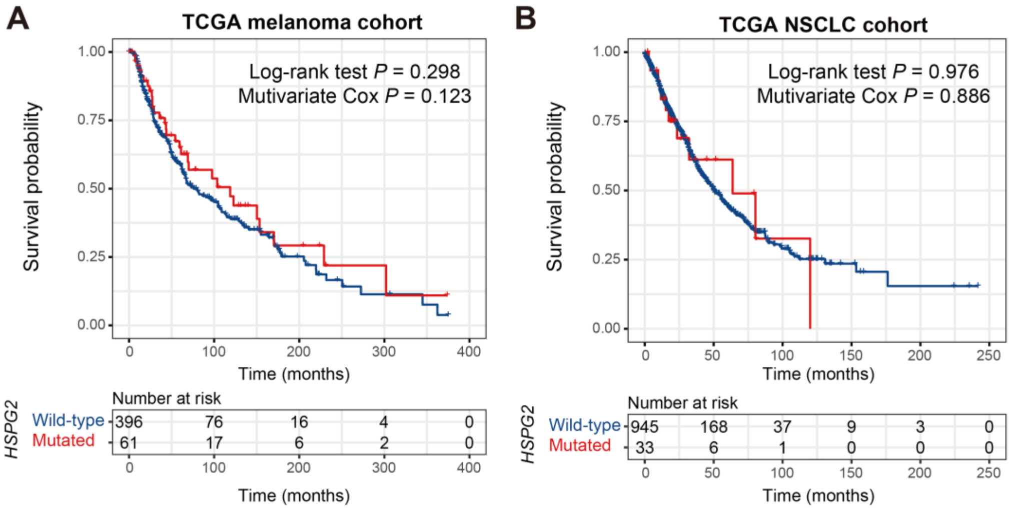

Figure S8. Prognostic capacities of *HSPG2* mutations in (A) melanoma and (B) NSCLC patients derived from the TCGA project.
